# ‘It's Powerful’ The impact of involving children and young people in developing paediatric research agendas: A qualitative interview study

**DOI:** 10.1111/hex.14028

**Published:** 2024-04-13

**Authors:** Laura Postma, Malou L. Luchtenberg, A. A. Eduard Verhagen, Els L. M. Maeckelberghe

**Affiliations:** ^1^ Beatrix Children's Hospital, University Medical Center Groningen University of Groningen Groningen The Netherlands

**Keywords:** agenda setting impact, children, child‐inclusive research, co‐researchers, impact, paediatric research agenda, priority setting partnerships

## Abstract

**Introduction:**

There is a growing consensus that children and young people (CYP) should be involved in matters that concern them. Progress is made in involving CYP in developing pediatric research agendas (PRAs), although the impact of their involvement remains unknown. We aimed to evaluate the impact of involving CYP in developing PRAs and assess the extent to which postpatient and public involvement (post‐PPI) activities were planned.

**Methods:**

We conducted a qualitative study using in‐depth interviews to identify and gain an in‐depth understanding of the impact of involving CYP in developing PRAs. The transcripts were uploaded to Atlas.ti to be coded and organised. Dutch‐language interviews were analysed and interpreted together with vocational education and training (VET) students. These students were aged between 14 and 18 years and were training to become nurses.

**Results:**

Three CYP and 15 researchers decided to participate. We focused on three categories of impact: agenda‐setting impact, individual impact and academic impact. Involving CYP creates a more enriched and clarified agenda. It ensured that both CYP and researchers underwent personal or professional growth and development, it created a connection between the people involved, awareness about the importance of involving CYP and it ensured that the people involved had a positive experience. The participants were unable to indicate the academic impact of their PRAs, but they did understand the key factors for creating it. In addition, the need to measure impact was highlighted, with a particular focus on assessing individual impact.

**Discussion:**

Our study outlines the diverse subthemes of impact that arise from involving CYP in developing PRAs. Despite the potential of research agendas to amplify CYP voices, only a minority of researchers strategized post‐PPI activities ensuring impactful outcomes, prompting the need for thorough evaluation of various impact forms and consistent alignment with the overarching goal of transforming the research field.

**Patient or Public Contribution:**

We involved VET students in the data analysis and interpretation phase by forming a young person advisory group. The data analysis of the interviews analysed by the VET students revealed four distinct themes: 1. Learnt new knowledge. 2. Learnt to collaborate. 3. Learnt to listen. 4. Assessment of the individual impact.

## 1 INTRODUCTION

There is a growing consensus that children and young people (CYP) should be involved in matters that concern them. Involving CYP is part of patient and public involvement (PPI). PPI is about ‘research carried out ‘with’ or ‘by’ members of the public rather than ‘to’ or ‘for’ them’.^1^ Flicker's research indicated involving patients is a necessary link between decision‐makers and potential knowledge users.^2^ By not involving the patients, researchers and clinicians may miss the needs the end users deem essential.^3^ Nowadays, therefore, CYP are involved in developing paediatric research agendas (PRAs) that were previously predominantly developed by researchers themselves.^4^, ^5^ A PRA refers to a systematic plan that outlines the research priorities related to the health of CYP.

The expected impact of involving CYP is crucial for justifying their involvement. Involving CYP is believed to contribute both to their personal development and to improve the research itself.^2^, ^6^ Most studies focus on describing PPI processes rather than exploring the impact of PPI.^7^, ^8^ Fortunately, there have been researchers who evaluated the impact of involving CYP and their families in paediatric research in general.^9^, ^10^, ^11^, ^12^ Vanderhout et al. found three different forms of impact involving CYP: impact on the research process, on the research team and on the CYP and their families.^11^ Other studies have shown that involving CYP in research impacted data analysis^10^, ^12^ and data dissemination.^10^ These studies are examples of evaluating the impact of involving CYP in paediatric research in general, not PRAs. Staley et al. evaluated the impact of involving patients in developing research agendas. They found that the impact of the evolving strength of the partnership itself and its capacity to influence others seems as important as the narrow product (the top 10 list) of a priority setting partnership.^13^ Recently, there have been calls to strengthen the evidence and to improve the methods for identifying, assessing, and recording the impact of PPI.^8^, ^14^, ^15^, ^16^ Not only does assessing the impact of involving CYP provide insight into the effectiveness of involvement but it is also needed to convince sceptics, justify the costs and increase funding.^17^ To meet these objectives, the Guidance for Reporting the Involvement of Patient and the Public checklist^18^ and the Public Involvement Impact Assessment Framework^19^ were developed. How these tools are used and how impact is reported, currently varies.^8^, ^20^

To explain the importance of evaluating the impact of involving CYP in developing PRAs instead of general research projects, it is important to acknowledge that the involvement of CYP in research can be divided into three phases: the preparatory, the execution and the translation phase. During the preparatory phase, CYP can be involved in the prioritisation of research topics,^21^ which involves developing PRAs. When CYP are involved in research projects, other than developing PRA, it can be categorised as the execution phase. Involving CYP in the translation phase means involving them in the dissemination, implementation and evaluation of research. The gap between the involvement of CYP in the execution phase on the one hand and the preparatory and translation phases on the other hand, remains.^4^ To assess whether CYP have an impact on the preparatory phase, it is necessary to evaluate the development of the PRAs in which they have been involved. This will help determine whether CYP involvement in developing the PRA was genuine or tokenistic.^5^ Many researchers remain dismissive of suggestions that patients should help prioritise research,^5^ risking tokenistic involvement.^22^ Furthermore, it is believed that the impact of CYP's involvement on stakeholders is as important as the impact on the PRA itself.^13^ This is another reason to evaluate the precise impact of involving CYP in developing PRAs.

The overarching goal for a PRA is to improve the overall health outcomes, addressing the priorities that impact CYP's wellbeing.^23^ Nowadays, there is increasing recognition of the necessity to plan for an additional phase of post‐PPI activities.^13^ However, often, the creation of the top 10 priorities list is seen as the final objective.^13^ This prompts the question of whether the intended goal of addressing the priorities is fulfilled. Hence, evaluating the impact of a PRAs is of utmost importance, to examine whether the overarching goal is met. Staley et al. found that it was unclear to researchers who developed a research agenda together with patients, who had the responsibility for keeping information up to date regarding the progress of the top 10.^13^ Staley et al. are the only ones who evaluated the impact of research agendas developed in collaboration with patients. However, CYP were not involved in the development of these agendas.

Previous research assessed the reported impact of involving CYP in developing PRAs. It was found that the impact on the PRAs was described in 41% of the agendas. The impact of the involvement of CYP on the CYP and researchers themselves was not described. In only 14% of the PRAs, post‐PPI action plans were described.^8^ The impact of involving CYP on the PRAs considered different research questions^24^, ^25^, ^26^, ^27^ and different priorities.^28^, ^29^, ^30^, ^31^, ^32^ In the second category, CYP had the same research questions but prioritised the questions differently than the researchers did. Despite the progress made in involving CYP in PRAs, once again this review shows that the impact is inadequately described.^8^ However, the absence of a description does not necessarily imply the absence of impact. Therefore, this study evaluates the impact of involving CYP in developing PRAs with CYP and researchers who developed a PRA together and assesses the extent to which post‐PPI activities were planned.

## 2 METHODS

We conducted a semistructured interview study to identify and gain an in‐depth understanding of the impact of involving CYP in developing PRAs and assess the extent to which post‐PPI activities were planned.

### 2.1 Participants

We aimed to include CYP and researchers experienced in developing PRAs together. Participants were identified via a literature review. Studies were included if they described the development of a PRA with the involvement of at least one CYP aged below 18 years and if the studies were written in English. This led to the inclusion of studies using three sources:

1. The systematic review of Odgers et al. Four studies were included that were published before October 2016.^4^
2. The narrative review of Postma et al. Twenty‐two studies were included that were published between October 2016 and March 2022.^8^
3. A literature search between March 2022 and August 2023 identified four more studies.

The researchers and CYP had to be Dutch or English‐speaking people. We contacted the first authors of the PRAs by email, and we sent all the participants an information letter and a consent form. If the first authors declined, we asked for the contact details of their co‐authors. In case of no response, we sent a first reminder after 3 weeks and again 3 weeks later if necessary. We requested the primary authors to reach out to the CYP who participated in their respective studies. Specifically, we asked the authors to inquire whether we could directly contact the CYP.

### 2.2 PPI

We involved vocational education and training (VET) students in the data analysis and interpretation phase by forming a young person advisory group. We believe that CYP from diverse backgrounds should be involved in research. We purposely involved VET students in our research because we aimed to listen to students who are not frequently involved in research. We contacted VET nursing schools and asked if the students were willing to be involved in our research in return for something meaningful to them, chosen by the students themselves. This led to extensive coordination with schools and a time‐intensive process before securing a school willing to participate.

The involved students were between 14 and 18 years old. Eleven girls and two boys volunteered to participate. We focused our analysis with the students solely on the Dutch‐language interviews on account of language barriers and time limitations faced by the students.^33^ Due to the time‐consuming recruitment process, the CYP were only involved in the data analysis and interpretation of this research. In return for their dedication to our research, the students were granted 2 days off from school to shadow in the hospital and visit a museum where we travelled through the human body.

### 2.3 Data collection

Regarding data collection and analysis, we followed the constructivist grounded theory approach developed by Kathleen Charmaz. This branch of grounded theory is used to create an interpretive representation of reality. The data were influenced by both the researcher and the participant because the researcher decides which questions are asked and interacts with the participants.^34^ We conducted semistructured interviews by teleconferencing to accommodate the geographically dispersed participants. The interviews lasted 1 h at most. A topic guide was developed based on the forms of impact described by other researchers^11^, ^35^ (File S1). Data collection and analyses occurred concurrently, allowing for iterative adjustments to data collection based on preliminary analyses. Insights emerging from early data shaped further data collection until no new insights emerged, aiming for theoretical saturation. The interviews were audio‐recorded and fully transcribed by an external company.

### 2.4 Data analysis

The transcripts were uploaded to Atlas.ti Mac (version 22.0.6.0) to be coded and the codes to be organised into themes and subthemes. First author L. P. coded the transcripts and discussed them with the research team. The analysis consisted of several steps, beginning with becoming familiar with the transcripts. Open coding was then applied to identify concepts and themes within the data. The next step involved axial coding, where we divided the codes into groups. This process involved constant comparison of the data to identify similarities, differences and patterns across interviews. We used data source triangulation to cross‐verify information from multiple sources. Instead of relying on a single data source, we gathered information from researchers and CYP to strengthen our conclusions.^36^

## 3 RESULTS

### 3.1 Participants

Out of the 35 persons we invited to participate (three CYP and 32 researchers), 13 did not respond, despite two reminders, and four decided not to participate because of time limitations, or because they were not working with children. Eighteen participants decided to participate, three of whom were CYP (17%) and 15 were researchers (83%) (Table 1). The CYP were between 17 and 24 years old at the time of their involvement. The participants represented six different countries from three continents. To guarantee participant anonymity, we only mention whether a quote originated from a researcher or a CYP. We were unable to include more CYP and because of these sampling constraints, we only achieved partial data saturation.

**Table 1:** Characteristics of the participants interviewed.

Characteristics	Young people (no.)	Adult researchers (no.)
Total	3	15
Sex		
Female	3	13
Male		2
Country		
Australia	1	2
Canada		2
Norway		1
The Netherlands	1	4
United Kingdom	1	5
United States of America		1
Work field		
Genetics		2
Nephrology		1
Oncology	1	3
Psychiatry		1
Psychology	1	2
Rheumatology	1	6
Methodology		
JLA	2	9
RPAC method		1
Workshop	1	2
Focus group		3

Abbreviations: JLA, James Lind Alliance; RPAC, Research Prioritisation by Affected Communities.

### 3.2 Categories

Based on the forms of impact identified in the literature, we focused on three categories of impact: agenda‐setting impact: the impact of the collaboration between CYP and researchers on the research agenda; individual impact: the impact of the collaboration between CYP and researchers on the researchers and CYP themselves; academic impact: the impact of the research agenda on newly published research. There is not yet a framework for what types of impact should be measured regarding PRAs; therefore; we used the categories of impact identified by others in general research projects.^11^, ^13^, ^35^ These categories were chosen because they are easily explainable and resonate with prior knowledge. The semistructured interviews allowed respondents to suggest additional categories or for the interviewer to ask further questions if a new category was introduced. However, this did not occur, so we did not add any new categories. The participants did elaborate on the need for measuring impact. Therefore, we added it as a secondary outcome of the research. This decision was also informed by the analysis of the VET students.

### 3.3 PPI

Three Dutch interviews were analysed with the VET students, one with a young person and two with a researcher. The data analysis of the interviews analysed by the VET students revealed four distinct themes: 1. Learnt new knowledge. 2. Learnt to collaborate. 3. Learnt to listen. 4. Assessment of the individual impact. We included the themes identified by the VET students in our analysis, occasionally making minor adjustments, while the meaning remained unchanged. For example, we divided new knowledge into new knowledge about research and new knowledge about the disease. We added the first three themes to individual impact and the last theme was added to the measurement and assessment section.

### 3.4 Agenda‐setting impact

Agenda‐setting impact was categorised by two themes: enrichment and clarification (Table 2). The quotes associated with focused impact are shown in Table 3.

**Table 2:** Themes of agenda‐setting impact.

Agenda‐setting impact	
Enrichment	Clarification
New perspectives	Ask for explanations—to researchers
New priorities	Ask for explanations—to children and young people
Additional notions	

**Table 3:** Quotes associated with agenda‐setting impact.

Agenda‐setting impact	Quotes
Enrichment	
Q1	‘So there was a lot of focus on how it [screen use] could be a waste of people's time and how it makes people anxious […] you know a lot of focus on that. Whereas the young people brought a more neutral or even positive approach. And that was a huge difference’. (RESEARCHER 7)
Q2	‘Yes, yes, they [CYP] indeed have a different set of questions, more about the future and their prospects. Those kinds of things were very important to them, whereas parents and researchers apparently haven't been thinking about them so much’. (RESEARCHER 1)
Q3	‘So, for instance, that was one of the things we [young people] brought to their [researchers] attention. That it was not about screen time, but about screen use in general’. (YOUNG PERSON 3)
Clarification	
Q4	‘I would question like, oh, what does this actually mean? Can you word it out? Like can you say not just this one word, but what that word actually means?’ (YOUNG PERSON 3)
Q5	‘So if we know that social well‐being is an important research priority area, what kinds of support resources or interventions are they looking for to improve whatever experiences they had or address any gaps in care that they experienced’. (RESEARCHER 5)

#### 3.4.1 Enrichment

The collaboration between researchers and CYP enriched the PRA in different ways. The CYP added new perspectives, new priorities and additional notions during the development of the PRA. New perspectives refer to different ways of looking at a situation. It involves adopting alternative viewpoints that were previously unconsidered or overlooked. New priorities refer to questions or topics placed on the agenda because CYP were involved. Additional notions refer to valuable understandings gained from CYP based on their ideas.

The CYP involved contributed to the refinement of existing concepts by offering new perspectives on certain ideas held by the researchers. One researcher mentioned that CYP held a more positive view of screen use, while researchers only focused on its negative side (Q1). Moreover, CYP made sure that new priorities that were important to them were added to the PRAs. They supplemented new priorities that differed from those of the researchers and parents involved (Q2).

Another subtheme that helped to clarify the PRA is that CYP provided researchers with additional notions. Unlike new priorities, where new questions or topics were brought forward, in this case, the existing topics and questions were refined. For example, CYP advised on what words to use to make the agenda more suitable for CYP (Q3).

#### 3.4.2 Clarification

The CYP played a significant role in enhancing agenda clarification. They posed critical questions to the researchers and sought clarification of the intended meanings of various research questions and the potential implications of researching a specific topic. Through such discussions, the CYP contributed to refining the PRA (Q4).

For agenda‐setting impact, it was not only considered important for CYP to ask for clarification but also for researchers. By involving CYP, researchers were allowed to question CYP about why they considered specific research questions, research outcomes or priorities important. In turn, this could lead to clarification of the research questions included in the agenda (Q5).

### 3.5 Individual impact

Involving CYP in developing a research agenda impacted the CYP and the researchers themselves. We called this individual impact. Five themes could be distinguished: personal growth and development, professional growth and development, connection between the people involved, awareness of the importance of PPI and positive experience (Table 5). Table 4 shows which themes emerged among CYP, the researchers or both.

**Table 4:** Themes of individual impact.

Individual impact				
Personal growth and development	Professional growth and development	Connection between people involved	Awareness of the importance of PPI	Positive experience
Empowerment (CYP)	*Improved communication (CYP)*	Meeting peers (CYP)	Awareness of the importance of involving CYP (R)	Enjoyable experience (CYP + R)
Taking myself seriously (CYP)	*Learn to collaborate (CYP* + *R)*	Altruism (CYP + R)	Knowledge of involving CYP (R)	Impressed by CYP (R)
Feeling included (CYP)	*Knowledge of research (CYP)*	Involved with patients (R)	Trust in research (CYP)	Changed dynamics in the team (R)
Increased self‐confidence (CYP)	*Knowledge of the disease (CYP)*			

*Note*: The subthemes that are presented in italics were developed together with the VET students.

Abbreviations: CYP, children and young people; PPI, patient and public involvement; R, researchers; VET, vocational education and training.

**Table 5:** Quotes associated with individual impact.

Individual impact	Quotes
Personal growth and development	
Q6	‘The oncologist who was there, he treated me in my hospital. So again, it was great to see him on the other side’. (YOUNG PERSON 1)
Q7	Translation: ‘I think that from that moment on, I kind of realised that: okay, there are adults who listen to me, and I can be taken seriously’. (YOUNG PERSON 2)
Q8	‘Because, as we know with this group, they suffer social isolation and that's a big problem. That was a big catalyst anyway for the research. So as soon as you make the young people feel included, you're already making such a difference’. (YOUNG PERSON 1)
Q9	‘So now I'll be in a meeting like we were in a massive meeting, there were big, big professionals and I was just chatting away [laughs]’. (YOUNG PERSON 3)
Professional growth and development	
Q9	‘I also learnt that I should express my opinion and engage in strategic debates at school’. (YOUNG PERSON 2)
Q10	‘I think I learnt that everyone can have such a different opinion, but if it's shared in the right way, then it doesn't matter. You can all sit there and share opinions. It might be completely different than someone else's but you have to listen to their opinion’. (YOUNG PERSON 3)
Q11	Translation: ‘I think that in everything I do, I am very aware of: what does someone think of it themselves? And that I am also aware of the fact that I cannot have a clear opinion about it, but that it is so important to also simply ask someone, what would you like yourself?’ (RESEARCHER 4)
Q12	‘And yet I just got a better idea of what the researchers are doing and kind of that side of things as well’. (YOUNG PERSON 1)
Q13	‘So I learnt a lot from the other young people, like different types of cancer and how that impacts, for example, fertility and how quickly they can get onto fertility support. A real big thing I learnt is about children can be admitted to the adult hospital and be treated’. (YOUNG PERSON 1)
Connection between the people involved	
Q14	‘I think we all built like that mutual connection at the end’. (YOUNG PERSON 2)
Q15	‘One of the groups I had, used to call me like “the favourite auntie that you never had”’. (RESEARCHER 10)
Q16	‘One girl's question was specifically about improving survival in brain tumors. And during that discussion, she had said, you know, I can see how that's important to me but it's not important to everybody else.‬ And so she took that question out of her top 10 because it was too specific’. (RESEARCHER 11‬‬‬‬‬‬‬‬‬‬‬‬‬‬‬‬‬‬‬‬‬‬‬‬‬‬‬‬‬‬‬‬‬‬‬‬‬‬‬‬‬‬‬‬‬‬‬‬‬‬‬‬‬‬‬‬‬‬‬‬‬‬‬‬)
Q17	‘But it does change their relationship all for the better, as well, sort of thing. So, you know, I learn much more about their lives and the impact of having whatever condition they have’. (RESEARCHER 6)
Awareness of the importance of PPI	
Q18	‘There is so much synergy that can happen when you put that kind of two parts of … two different areas of knowledge together. That's really powerful’. (RESEARCHER 5)
Q19	‘I have to say, I've been a researcher for over a decade and the patient priority setting exercise that we did was probably one of the most worthwhile research endeavors I've ever had the pleasure of being a part of’. (RESEARCHER 8)
Q20	‘Yeah. We tend to be so much in our own hats in doing research and just … it's just so easy not to think of them as human beings with actual … actual problems’. (RESEARCHER 15)
Q21	‘I guess you think more about why you're doing the work that you're doing and whether it is actually relevant’. (RESEARCHER 12)
Q22	‘What if they don't like the intervention that you have spent the last six months developing? If they say, no, we'll never use that, what are you going to do? You know, you should have involved them six months ago. I think, researchers aren't yet good at that sort of thing’. (RESEARCHER 6)
Positive experience	
Q23	‘At one point, the girls in the group started singing and had made a song out of whatever they were talking about [laughs]. And afterward, she [leading researcher] said I don't think I've ever done a focus group where the participants have burst into song. [laughs]’. (RESEARCHER 6)
Q24	‘We all had the same goal. And the word powerful is the only word I've got. It's powerful. It was so powerful’. (RESEARCHER 8)
Q25	‘I was really impressed about how they discussed it and listened to each other's… you know, if there was one not in the top five and someone would say, oh, I think this is important, could we move it up? And they would think about which one needed to come out’. (RESEARCHER 11‬‬‬‬‬‬‬‬‬‬‬‬‬‬‬‬‬‬‬‬‬‬‬‬‬‬‬‬‬‬‬‬‬‬‬‬‬‬‬‬‬‬‬‬‬‬‬‬‬‬‬‬‬‬‬)

#### 3.5.1 Personal growth and development

Being involved in PRAs had several advantages for the personal growth and development of CYP. They experienced a feeling of empowerment by finding themselves in the company of peers who had gone through similar experiences and seeing their physicians in settings other than their surgeries (Q6). They were grateful for the opportunity to share their thoughts. By engaging in discussions about what was important to them, CYP felt that they were respected and taken seriously, and this taught them to take themselves seriously as well (Q7).

The CYP mentioned that they felt welcomed and supported by the research group and felt part of the group. One young person mentioned that if researchers made the CYP feel included, it would contribute towards reducing social isolation (Q8). All CYP mentioned that their confidence had grown during the collaboration. They had all been shy initially, but later in the project, they felt comfortable about participating in the discussions (Q9), although it did depend on the people who ran the collaboration to make the CYP feel confident.

#### 3.5.2 Professional growth and development

Involving CYP had several advantages for professional growth and development, both for CYP and researchers. One subtheme was improved communication. Through their involvement in developing the agenda, CYP had the opportunity to communicate their opinions and ideas. They mentioned that the communication skills they had learnt, thanks to their involvement, stood them in good stead during school activities (Q9).

The CYP noticed that they had learnt to collaborate with different team members even though they did not share the same opinion (Q10). Researchers mentioned that they had learnt to collaborate with the CYP without experiencing power imbalance. A researcher, who is also a physician, said that because of their partnership, he has changed how he interacts with CYP in his surgery and now involves them more in decision‐making (Q11).

Furthermore, through their involvement, CYP gained insights into the work of researchers. This deepened their understanding of how research advances knowledge and addresses questions (Q12). Finally, the CYP mentioned that they had gained knowledge about their diseases. For example, they recognised that individuals could experience the same disease in different ways and that even researchers and medical doctors do not have all the answers (Q13).

#### 3.5.3 Connection between the people involved

Enduring relationships developed between participants that might continue after PRA development officially ends, thanks to social media or by working together on another project. This was a noteworthy result of the collaboration. The CYP mentioned that the collaboration contributed to the connection between peers suffering from the same disease. They could share their stories, make new friends, have dinner with other young people and enjoy activities together with their peers during the priority‐setting meeting (Q14/Q15).

Another phenomenon the researchers noticed that contributed to the connection between the people involved was the altruistic attitude of the CYP. They were all aware of framing their questions as generally as possible, so it would capture many issues in one (Q16). Researchers and medical doctors felt that their relationship with CYP had changed for the better. They felt more connected to each other after developing the PRA together because they learnt about what is important for CYP and what the impact is of having a certain disease (Q17).

#### 3.5.4 Awareness of the importance of PPI

Researchers came to understand the importance of incorporating CYP in determining research priorities. They had a greater appreciation for CYP's contribution to establishing research objectives because of their increased awareness of the insights and viewpoints CYP had to offer. Even researchers who already had positive thoughts about involving CYP in developing research priorities mentioned that now they appreciated even more how special it is to collaborate with CYP. They felt fortunate to be in a group with CYP and realised that other groups do not have such an advantage (Q18/Q19).

The attitudes and motives of researchers and other professionals involved, such as paediatricians, nurses, and dietitians, were significantly impacted by the inclusion of CYP in the establishment of PRAs. Engaging with young people forced practitioners to consider the importance and relevance of their work and consider whether it met the needs of the people they were trying to help. Physicians were more aware of the impact a disease had on their patients, while researchers were more aware that their ‘research subject’ was an actual human being (Q20/Q21).

Researchers noticed that they had gained knowledge about how the CYP wanted to be involved in developing research together. They had learnt that young people should be involved from the start, preferably as part of the steering committee. Researchers had learnt from other colleagues, who were used to involving CYP in their research, but also from the CYP, who had clearly expressed how they would like to be involved (Q22). Finally, involving CYP strengthened the trust in the research process through the openness and inclusivity of the conversations between researchers and CYP. The CYP established trust in the research community by realising that their opinions were appreciated and heard. Researchers were aware of how important it was to gain the CYP's trust by involving them as equal partners in the development of PRAs.

#### 3.5.5 Positive experience

Involving CYP in developing PRAs created a positive experience. First, involving CYP in PRA development created enjoyable experiences for all participants involved. Involving CYP and their contributions brought energy and enthusiasm. The participants were encouraged to work together, to be creative and to show enthusiasm for the collaboration. This made defining the agenda a memorable and worthwhile experience for everyone (Q23).

First, one enthusiastic researcher mentioned that at the end of the day, it was a feeling like no other because they had accomplished something that would have tangible results (Q24). Second, the numerous relevant thoughts and insights that CYP shared with researchers impressed them. The CYP were very keen on getting their priorities right and were able to sort that out amongst themselves, discussing, deliberating and weighing the arguments (Q25). Third, involving CYP significantly changed the dynamics of the research team that developed the PRAs. The power dynamics that were connected to adult‐led decision‐making processes had changed, resulting in a more inclusive setting.

### 3.6 Academic impact

We noticed that CYP had no knowledge of or updates on what had happened to the PRA after it had been published. Researchers were hardly aware or did not actively keep track of whether new studies based on their PRAs were being published. None of the research teams had initiated literature searches in their area since they had published their PRAs. Most of them were aware through word of mouth that the research priorities were being studied. Occasionally, some researchers noticed that the agenda had been referenced by others (Q26).

In retrospect, most researchers acknowledged that they should have given more thought to how they wanted to implement and keep track of their PRAs. One researcher mentioned that elaborating on the priorities and creating collaborations was of utmost importance, not necessarily the publication itself (Q27).

The participants were unable to provide a clear indication of the academic impact of their PRAs. Nevertheless, they did gain an understanding of what was important when planning post‐PPI activities and creating academic impact. These aspects are divided into three groups: awareness and traceability, help from the research team and help from other organisations (Table 7). The quotes associated with academic impact are shown in Tables 6 and 8.

**Table 6:** Quotes associated with academic impact.

Academic impact	Quotes
Q26	‘It [PRA] is still being used and I can see in papers, you know, when people say this area is important as identified by, and they quote our work’. (RESEARCHER 6)
Q27	‘Who cares if it's been published? It's how we're going to use them, you know? The publication is a vehicle, not necessarily our endpoint’. (RESEARCHER 8‬‬‬‬‬‬‬‬‬‬‬‬‬‬‬‬‬‬‬‬‬‬‬‬‬‬‬‬‬‬‬‬‬‬‬‬‬‬‬‬‬‬‬‬‬‬‬‬‬‬‬‬‬‬‬‬‬‬‬‬‬‬‬‬)

**Table 7:** Themes of academic impact.

How to create academic impact		
Awareness and traceability	Help from the research team	Help from other organisations
Creating awareness of the agenda	Stay involved in the research agenda	Political attention
Visibility of the agenda		Funding for agenda
Specific research questions		Funding for research questions

**Table 8:** Quotes associated with academic impact—awareness and traceability, help from the research team and role of other organisations.

Academic impact	Quotes
Awareness and traceability	
Q28	‘It is a research question, so there are indeed different aspects to consider, and I think we have managed to capture them quite well. The questions are fairly clear in my impression, and the involvement of all the different parties was simply essential for that’. (RESEARCHER 3)
Help from the research team	
Q29	‘It would be great to have another opportunity to vocalise the things that did not make the top three, I would feel more validated, and I feel like it could make for really good change in the industry, and I feel like the conversation is not finished like we still need to discuss more’. (YOUNG PERSON 1)
Role of other organisations	
Q30	‘One thing that remains challenging is that you can create a wonderful top ten list. However, it is also uncertain how the politics are positioned on this matter. We may know exactly what the children and other participants consider important, but what is the status of funding from the political side?’. (RESEARCHER 2)
Q31	‘So we [funding agency] want to fund a paediatric study, can you [researcher] suggest any so then we can use this to say “well, this is the top priority of maybe these top three or one of these top ten”. So the funding agency asked the chairs of our research team, where should we be putting our money? And then they put a commission call’. (RESEARCHER 9)
Q32	‘If there was evidence out there to say if this is on the research priority list, it's got a 10‐fold more likely chance of getting funded’. (RESEARCHER 9)

#### 3.6.1 Awareness and traceability

All respondents agreed that consideration should be given to how awareness of the agenda would be ensured before developing the PRA. Researchers also mentioned that it was important to think about how people were going to find the agenda when it was ready to be used by others. Suggestions to ensure the visibility of the agenda included: publishing the PRA open access or creating a website where the agenda could be published (e.g., the James Lind Alliance website), including new studies based on the priorities.

Participants noted the importance of formulating specific research questions instead of broad themes. Researchers and CYP, who had identified themes instead of research questions, expressed difficulties in monitoring whether a theme had been sufficiently addressed or answered. One researcher emphasised how important it was to involve all relevant stakeholders when formulating comprehensive and specific research questions (Q28).

#### 3.6.2 Help from the research team

To make sure the PRA had academic impact, participants mentioned the importance of the research team staying involved, especially after the agenda was published. They could take on one of the research priorities themselves, but more importantly, the research team should enable a widespread implementation to make academic impact. Furthermore, one of the young people considered it important to take responsibility for advocating priorities not included in the agenda (Q29).

#### 3.6.3 Role of other organisations

Academic impact was also considered achievable through other organisations. Examples mentioned by the participants: agencies that helped fund the development of the agenda, community agencies or patient associations that created attention for the agenda and agencies that funded the elaboration of the priorities from the agenda. To stimulate innovation and address social concerns, the government should prioritise and invest in establishing the PRAs (Q30). Some researchers mentioned that funding agencies eagerly awaited the completion of the PRAs so they could ensure that their financial resources were directed towards projects that held the most potential for valuable contributions to the patients. Another researcher mentioned that every research team should be connected to a funding agency so these agencies could recommend the use of the priorities on the agenda (Q31).

Most of the respondents encountered difficulties in securing funding for their published priorities. The implementation of the agenda could also be quite challenging even though it was developed together with CYP. One researcher mentioned that measuring the academic impact of the PRA might also help to obtain funding for developing the agenda in the first place (Q32).

## 4 MEASURING IMPACT

A secondary outcome of this research was the participants' views on the need for measuring the different types of impact. Quotes associated with the need for measuring impact are shown in Table 9.

**Table 9:** Quotes associated with the need for measuring impact.

Measuring impact	Quotes
Assessing individual impact	
Q33	‘The partnership helped to form some friendships and people stayed in touch post the work … so this is not a play in the outcome, but it is a good one and probably on of the most important’. (RESEARCHER 13)
Q34	‘And I feel like even if it's just that feeling of mutual connection like that's enough for me to be like this is making an impact because I'm impacted knowing that I'm not alone. And for me that's enough’. (YOUNG PERSON 1)
Q35	‘I think if we measure everything all the time, then we begin to lose the authenticity of the project’. (YOUNG PERSON 3)
Assessment of academic impact	
Q36	‘Some people perceive those priorities that are less kind of medical as not important, there is a need for a shift in this thought […]. Measuring whether the outcomes of the agenda are used could help to evaluate this shift’. (RESEARCHER 12)
Q37	‘I haven't heard what actually happened with the paper. It'd be really good to know its impact. I guess that's unfortunately with the research, like once the paper is published. It's the gap between that and clinical’. (YOUNG PERSON 1)

### 4.1 Assessing agenda‐setting impact

Researchers advocated for assessing the agenda‐setting impact of involving CYP for each PRA. For this to work, they recommended transparent reporting of the impact the CYP had on the agenda, so it would be obvious which priorities originated from the CYP. The reasons for assessing the agenda‐setting impact were diverse. For example, knowing what the impact was of involving CYP in agendas would make it easier to obtain funding or convince sceptical team members to involve CYP.

### 4.2 Assessing individual impact

While, according to the participants, the research outcomes were valuable, the positive impact on CYP and the researchers involved were even more important to them. Multiple researchers mentioned that if CYP felt empowered, were heard, returned and had fun, that was a sufficient measure of impact for them. The CYP believed it was crucial to inform researchers about what the ongoing issues were for them, and the CYP trusted the researchers to initiate new projects about that matter. One young person mentioned something similar. She felt that the sense of connection with other peers was more important than the actual impact on the PRA (Q33/Q34).

On the one hand, a researcher mentioned that it would be impossible to quantify and measure the individual impact of involving CYP. She proposed to qualitatively evaluate the impact of involving CYP, which should be the responsibility of each research team after publishing the PRA. On the other hand, a young person concluded that if researchers had to measure the impact of CYP's involvement, it would cause more stress and reduce authentic outcomes (Q35).

### 4.3 Assessment of academic impact

Overall, researchers were unsure about which specific categories of impact should be measured. One researcher mentioned that the academic world sets a great store by publishing articles or presenting abstracts at conferences together with CYP, while for most of the researchers, those activities do not contribute to impact. The true worth of the collaboration lay in the moment when individual impact was reached. Still, they agreed that sometimes it might be beneficial to measure or assess the academic impact of involving CYP because it may be required by funding agencies or simply to check whether the priorities are used and the goal of changing the research field is reached (Q36/Q37).

## 5 DISCUSSION

This study evaluated the impact of involving CYP in developing PRAs. We found two subthemes for agenda‐setting impact and five for individual impact. By identifying these subthemes, we offer a detailed insight into the diverse contributions the CYP bring, specifically for PRAs. We found that only a minority of researchers strategically organised post‐PPI activities to guarantee academic impact. However, they gained an understanding of some key factors to consider when planning these activities and achieving academic impact.

We found that involving CYP in developing a PRA had an impact on the agenda by enriching and clarifying it. This was in line with a scoping review published by Vanderhout et al. They found that the impact of patient and family involvement included enhancing the usefulness or relevance of the findings in general research projects.^11^ Staley et al. found that translating a top 10 priority into a research project has three steps, including developing a focused research question from the broad priority.^13^ Ensuring well‐defined research questions in collaboration with CYP may have the potential to accelerate the adoption of these questions by other researchers (Postma et al., submitted). When research questions are formulated, funders and researchers can identify which questions warrant subsidies.^13^ This might reduce research waste by directing funding to questions of genuine significance.^5^

Individual impact was regarded as an important form of impact, despite that this category is often being overlooked.^8^ When considering individual impact, only the positive aspects are examined, yet the negatives need to be considered as well.^37^ In the definition of impact proposed by the National Institue of Health and Care Research (NIHR) (benefits and learning gained from the insights and experiences of patients, carers and the public when working in partnership with researchers),^38^ there is no mention of negative impact. Nevertheless, the NIHR national standards for public involvement recognise that there is valuable learning from both positive and negative impacts. Therefore, it is important to evaluate the individual impact, including the negative aspects.^30^

The importance of the need for measuring impact was highlighted during the interviews. It was widely acknowledged that it is crucial for the agenda to yield tangible outcomes, evaluating these outcomes may be a necessary step for ensuring that the voices of CYP are heard and the research field is changed. However, participants mentioned the complex nature of measuring the impact of involving CYP in PRAs. This is also found in previous studies evaluating the need for measuring the impact of the involvement of CYP in general research projects.^15^, ^17^ Therefore, we should be cautious to not oversimplify measuring the impact of involving CYP because PPI is a complex social process and therefore difficult to quantify.^17^, ^39^ In the medical field, quantifiable metrics are commonly used to measure impact, for example when measuring the impact of journals using quantifiable metrics such as impact factor.^40^ Evaluating the impact of involving children requires a shift towards qualitative evaluation methods. Therefore, it was suggested to adopt qualitative approaches to evaluate the impact of involving CYP, acknowledging the unique complexities involved in this context.

### 5.1 Strengths and limitations

The strength of our study was the inclusion of participants from three different continents. This diversity enriched the data and enhanced the generalisability of the findings. Our study had several limitations. First, the interviews challenged the participants to recollect prior encounters with PPI, some of which went back many years. Possibly the data would have been more extensive if the evaluation had been conducted immediately after completion of their study.^40^ Second, although we aimed to interview more CYP, we only included three. The reason was that the studies in which CYP were involved had already been completed, the collaboration ended and the CYP had not provided consent to share their contact details beforehand. Furthermore, the involvement of CYP in developing PRA is limited, so the source was not exhaustive. Only reaching partial data saturation might lead to the underrepresentation of the CYP's perspective. These limitations highlight the importance of measuring the impact of PPI timely as the project is still fresh in memory and all the team members are still involved, allowing for the inclusion of everyone rather than just a few stakeholders.

### 5.2 Implications and future research

Our results offer valuable insights for the development of guidelines for involving CYP in developing PRAs or for creating checklists to measure impact. Incorporating these impact subthemes can be indicative of effective involvement. It is crucial to acknowledge the fluid nature of different forms of impact, allowing for the inclusion or removal of subthemes as needed, based on their continued relevance and contribution to the participatory process. The reason for regarding individual impact as more important than the agenda‐setting impact might be that only researchers and CYP were interviewed. Funders of the research agenda, policymakers or representatives from health organisations and nonprofit organisations advocating for the interests of CYP might have different opinions. Further research into the opinions of these stakeholders is therefore important. The limitations of this study emphasise the importance of timely measurement of the impact of PPI. This might make it easier to assess the impact, as the project will be still fresh in people's minds. Furthermore, all the team members are still involved, allowing for the inclusion of everyone rather than just a few stakeholders. Therefore, we recommend integrating the evaluation of impact as a standard component of developing PRAs involving CYP. We advise the JLA to address this point in the guidelines for developing research agendas.

## 6 CONCLUSION

Our study provides an overview of the various subthemes of impact that arise when involving CYP in developing PRAs. This understanding can lead to tailored approaches and strategies to enhance the impact of CYP in PRAs. It is worth noting that only a small number of researchers planned post‐PPI activities to ensure the agenda had an impact. Research agendas can be a useful tool in achieving the goal of changing the research field and amplifying the voices of CYP. However, it is important to consider whether this goal is always achieved. To ensure success, it is crucial to evaluate the different forms of impact and remain mindful of the ultimate goal of changing the research field.

## AUTHOR CONTRIBUTIONS

**Laura Postma**: Conceptualisation; investigation; writing—original draft; methodology; visualisation; writing—review and editing; formal analysis; project administration; software; data curation; validation; funding acquisition; resources. **Malou L. Luchtenberg**: Writing—review and editing; supervision; conceptualisation; methodology; formal analysis; resources. **A. A. Eduard Verhagen**: Conceptualisation; writing—review and editing; methodology; supervision; formal analysis; resources. **Els L. M. Maeckelberghe**: Methodology; conceptualisation; writing—review and editing; supervision; formal analysis; resources.

## CONFLICT OF INTEREST STATEMENT

The authors declare no conflict of interest.

## ETHICS STATEMENT

The medical ethics review committee of University Medical Center Groningen concluded that our study fell outside the scope of the Dutch Medical Research Involving Human Subjects Act. The data were anonymised and personal information was only available to the research team. The participants were entitled to end their participation at any time. The participants did not receive a financial reward for participating. The anonymised transcripts of the interviews shall be saved for 5 years and all audio recordings were destroyed to protect the privacy of the participants.

## Supporting information

Supporting information.

## Data Availability

The data that support the findings of this study are available on request from the corresponding author (Laura Postma). The data are not publicly available due to privacy or ethical restrictions.
