# Supplementary material for: ‘It's Powerful’ The impact of involving children and young people in developing paediatric research agendas: A qualitative interview study
Source: Health Expect. 2024 Apr 13;27(2):e14028. doi: 10.1111/hex.14028 (PMC11015888; doi:10.1111/hex.14028)
Supplement: Supplementary file 1 — Supporting information. [file HEX-27-e14028-s001.docx]

**Supplementary file 1: Topic Guide**

1. Topic guide for interviews with researchers

| Topic | Questions: |
| --- | --- |
| Method | 1. Can you please tell me something about how you developed the research agenda?    - How did you do this?    - Can you tell me why you chose this method to develop a research agenda?    - I am not quite sure what you mean by … ? 2. How did you involve children in developing the research agenda?    - How did you involve children of different ages?    - Can you give an example of…?    - Can you elaboratie on ...?    - Can you describe a situation in which this was not the case…?    - Before you mentioned that … now you are saying … can you explain this? 3. What criteria have to be met to consider a method, a qualitative good method to involve children in developing a research agenda?    - Why do you think this criterion is important?    - If this criterion is not met, do you consider the method then as qualitative poor? 4. How would you prefer to involve children in developing a research agenda?   **Summary of this section.** |
| Project impact | 1. Can you tell me how the research agenda was created?    - What impact did the children have on it?    - How can you say children had an impact on the research agenda? |
| Individual impact | 1. Can you tell me how you experienced involving children in developing a research agenda?    - You are now mentioning positive experiences, did you have any negative experiences when involving children?    - Did the involvement of children have an impact on you?    - Do I understand you correctly when I say that … |
| Research impact / Long term impact | 1. What has happened since you published the research agenda?    - How did you make sure the research agenda is visible for others? |
|  | 1. There is a lot of discussion about whether the impact of the involvement of children should be measured, I am very curious what your opinion is regarding that topic |
|  | 1. Do you want to add something to this interview, what you consider as important?   **Summary and thank participant.** |

| Topic | Question |
| --- | --- |
| Method | 1. Can you please tell me something about the research of … in which you were involved?    - Can you tell me something about how you experienced working together with researchers?    - Can you tell me something about how you experience developing a research agenda? 2. Can you tell me something about the way young people were involved in the project?    - What do you think about this method?    - These are all positive things, are there also negative things about this way of involving young people?    - Can you give examples of that? 3. If you could decide everything in research, how would you prefer to be involved? Of if you could involve young people in developing a research agenda, how would you do that?    - Why do you think this is important?   **Summary of this section.**  **.** |
| Project impact | 1. Do you think you had an impact on the top three priorities?    - Why do you think that?    - How did you manage to make an impact?    - Do you have tips for other young people on how to make an impact on the top three? |
| Individual impact | 1. Did your involvement have an impact on you? 2. Did you learn something about being involved in this project? |
| Research impact/ Long term impact | 1. Do you know what has happened to the top 3 priorities? 2. Did you hear anything about the project? What do you hope happens to the top 3? |
|  | 1. Do you want to add anything that you think is important? That we haven’t discussed yet?   **Summary and thank participant** |

1. Topic guide for interviews with children and young people
